# 

**Published:** 1813-12

**Authors:** 


### MONTHLY CATALOGUE OF MEDICAL BOOKS.

SYNOPSIS Nosologiæ Methodicæ Auctore Gulielmo Cullen, M.D. To which is added an Appendix, containing a Synopsis of the Systems of Sauvages, Linnæus, Vogel, Sagar, M'Bride, Cullen, Swediaur (1812), Young (1813), and a translation of Cullen's Nosology, with references to the best authors who have written since his time. By John Thomson, M.D.—Longman and Co.

Description of the Retreat, an institution near York, for insane persons of the Society of Friends; containing an account of its origin and progress, the modes of treatment, and a statement of cases. By Samuel Tuke.—Darton and Harvey.

Lectures on Inflammation, exhibiting a View of the general Doctrines, pathological and practical, of Medical Surgery. By John Thomson, M.D. F.R.S.E. Professor of Surgery to the Royal College of Surgeons, and Regius Professor of Military Surgery in the University of Edinburgh.—Cadell and Co.

Medical Transactions, published by the College of Physicians in London. Vol. IV.—Longman and Co.

Observations on the Nature and Treatment of Consumption; addressed to Patients and Families. By Charles Pears, M.D. F.L.S. 8vo.—Highley and Son.

Anatomical Tables of the Human Gravid Uterus; in Two Classes. By Mr. Hogben, Surgeon. Dedicated to the Royal College of Surgeons, London.—Callow.

A Treatise on Midwifery; containing References to the Anatomical Tables.—Callow.

---

### *NOTICES TO CORRESPONDENTS.*

*The Editor is much obliged to his friend Dr. J. C. Warren, for his care in supplying him with the New England Medical Journal, the two last numbers of which have just been received. As the London Medical and Physical Journal finds its way to Boston when letters are intercepted, Dr. W. must accept a public instead of a private acknowledgment, for his kind offices to the Editor on various occasions.*

*A physician wishes to be informed by the author of the Memoir on Medical Reform, whether, when he says "The Edinburgh College of Physicians are obliged to admit all Doctors of the Scottish Universities to their association without examination, and only on paying the necessary fees;" he means to state, that those Doctors who have obtained their degrees in physic from Aberdeen or St. Andrew's by certificate, can claim admission into the Edinburgh College, on paying the necessary fees.*

*The names of several gentlemen have been received as subscribers to the General Index, and others are solicited.*

*Want of room has occasioned the insertion of communications from the following gentlemen to be deferred till our next Number:—Dr. Young; Mr. G. Borrett; Mr. C. Abel; Mr. Robert Semple; Mr. L. W.; W. M. T.*

*The back Numbers will continue to be sold at* 1s. 6d. *till February* 1, *as announced in our last.*

---

### *CORRIGENDA.*

| | | | |
|---|---|---|---|
| *For* Dr. Yeates, | p. 465 | *read* | Dr. Yeats. |
| — Viq. d'Azyr, | p. 501 | — | Vicq. d'Azyr. |
| — Ditto, | p. 502 | — | Ditto.

### INDEX

### TO THE THIRTIETH VOLUME.

ABEL, Mr. his translation of Portal's Memoirs on Apoplexy, 193, 370
Abstinence, extraordinary, remarks upon 103
Acids, influence of, on the composition of urine 328
Adams, Mr. W. on the cure of ophthalmia 46
Adams, Dr. on inflammation in hydrophobia 201
Agate, action of, on light 427
Agriculture, influence of, on man 13
Air, inflammable, explosion of 427
Albuminous concretion in the cavity of the thorax, account of 73
Alcohol of sulphur, account of 80
Alcohol, on the existence of, in fermented liquors 392
Anatomy, studied by the Rajah of Tanjore 219
Anatomy and physiology, progress of 6
Animal chemistry, progress of 8, 9, 12
Animals without vertebræ, account of 430
Apoplexy, on the nature and treatment of 193, 370
Apothecaries and surgeon-apothecaries, objects of their association 3
Apothecary, remarks on the profession of 48
——————— on the qualifications of 467
Armstrong, Dr. John, on brain fever 77
Arsenic, utility of, in herpetic affections 23
———, its *modus operandi* 27
———, use of, in cutaneous complaints 296
Atkinson, Mr. James, case of tumor on the neck 353
Atropa belladona, effect of, in dilating the pupil 69
Baths, warm, establishment of, at Paris 223
Barytes, carbonate of, utility in consumption 466
Bateman, Dr. T. on cutaneous diseases 412
Bats, observations on the tribe of 429
Bellingham, Mr. on hepatic and pneumonic inflammation 190
Berard, J. E. on the specific heat of gases 399
Berzelius, Dr. on the composition of blood 9, 10
————————, result of his analysis of bile 12
Bill, apothecaries, opposition to, by the colleges of physicians and surgeons, 5; remarks upon 479
——— for preventing the small-pox 171
Blane, Sir Gilbert, on marsh miasmata 15, 160
Blood, composition of 9
——— vessels, art of injecting 89
BOOKS, NEW MEDICAL, &c. CRITICAL ANALYSIS OF.—Edinburgh Medical and Surgical Journal, No. xxxiii. 68. Ditto, ditto, No. xxxiv. 245, 337. No. xxxv. 506. Corvisart on the Diseases and Organic Lesions of the Heart and Great Vessels, 141. Ward's Account of Mary Thomas and Anne Moore, 157. Medico-chirurgical Transactions, Vol. iii. 159, 283. Dr. Gibney on Sea-bathing, 224. Johnson on the Influence of Tropical Climates, 227. Coley on the Remittent Fever of Infants, 237. Sutton's Tracts on Delirium Tremens, &c. 333. White on contracted Intestinum Rectum, 403. Bateman's Synopsis of Cutaneous Diseases, 412.
Books announced 88, 176, 264, 347, 352, 439, 528
Boringdon, Lord, bill presented by, for preventing the small-pox 171
Botanical report 524
Brain, treatment of, with alcohol 56
———, desiccation of 57

Brain, constituents of, soluble in alcohol 63
Brande, W. T. on magnesia 323
——, on acids in calculous complaints 324
——, on alcohol in fermented liquors 392
Bree, Dr. remarks on splenitis 169
Burell, Dr. case of scirrhus in the intestines 515
Buxton, Dr. his treatment of coughs objected to 49
Calculous complaint relieved by magnesia 325, 326
Carditis, cases of 151
Cases—Of herpes, 23, 26; of obstructed intestine from spasm, 29; of spasm of the uterus, 31; of small-pox after vaccination, 33; of convulsions during labour, 36; in which resuscitated salivation was beneficial, 37; of hernia cerebri, 74; of dysentery, 75; of vomiting, 100; of extraordinary abstinence, 104; of insanity, 125, 434; of sub-pericarditis, 143; of adhesion of the pericardium to the heart, 145; of conversion of the muscular tissue of the heart into a cartilaginous substance, 147; of contraction of the left auriculo-ventricular aperture, 148; of carditis, 151; of rupture of the heart, 153; of perforation of the septum of the ventricles, 155; of ovarian dropsy, 162; of diseased testicle, 164; of difficult parturition, 166; of cynanche laryngea, 166; of hepatitis, 190; of cutaneous affections, 297; of labour pains, 303; of rupture of the uterus, 316, 444; of abdominal pregnancy, 318; of typhus fever, 321; of calculus, 325, 326, 328, 330; of delirium tremens, 334; of hæmatirrhœa, 329; of tumor on the neck, 353; of Ann Foulkes, 355; of rheumatism, 363, 367; of apoplexy, 371; of contracted rectum, 404; of peritonitis, 409; of constipation, 441; of puerperal convulsions, 455; of urinous vomiting, 465, 493; of pulmonary consumption, 466; of periodical day blindness, 509; of epilepsy, ib.; of injury to the fœtus without the mother being affected, 510; of cold applications to ulcers, 511; of hæmatemesis, 513; of scirrhus in the intestines, 515.
Cataract, operation for, assisted by hyoscyamus 71
Cerebral matter of man, analysis of 55, 66
Chemistry, progress of 9, 12
Chevalier, Mr. on ovarian dropsy 162
Children, Mr. account of his galvanic battery 426
Climates, medical effects of 129
Clutterbuck, Dr. examination of his opinion that all diseases are local 180
Cold, application of, useful in peritoneal inflammation 19
—— only the predisposing cause of mortification 20
Coley, Mr. J. M. on the remittent fever of infants 237
—————— on urinous vomiting 465
Collectanea Medica 55, 129, 207, 323, 392, 499
Colleges of physicians and surgeons, contrast between 275
Collingwood Main Colliery, explosion of inflammable air in 427
Collins, Mr. on opium in labour pains 303
Committee, London, of apothecaries, proceedings of 3, 4, 307, 312, 473
Committee for examining Messrs. Delahoyde and Lucett's process for curing insanity, proceedings of 433
Consumption, cured by carbonate of barytes 466
Convulsions during labour cured by brandy and water 36
——————, puerperal, remarks upon 111, 455
Cooke, Mr. dissection of a cancerous stomach 337
———— on hæmatemesis 513
Corvisart, Dr. J. N. on diseases of the heart 141
Cough, remarks on Dr. Buxton's treatment of 49
Cutaneous diseases, synopsis of 412
——————, definitions of 414
——————, arrangement of 416
Cynanche laryngea, case of, with the dissection 166
Davies, M. D. H. on mercurial action in diseases 446
Deaths by small-pox 522
Delahoyde, Mr. his mode of treating insanity 434
Delaroche, Dr. on radiant heat 396
————, on the specific heat of gases 399
Delirium tremens, account of 17, 333
———— cured by the use of opium 17

Delirium, case of 334, 335
Diseases, division of, into general and local, inquiry respecting 180
———, monthly report of 438, 528
O'Donnell, Dr. on hydrophobia 19
Douches, establishment of, at Paris 223
Dropsy, ovarian, case of 162
Drugs, prices of 437, 526
Dunn, Mr. on extraordinary abstinence 103
Dysentery, cured by ipecacuanha and laudanum 75
Earl, Mr. Henry, on diseased testicle 164
English, Mr. W. case of injury of the fœtus without the mother being affected 510
Eye, pupil of, dilated by belladonna and other narcotics 69
———, effects of hyoscyamus upon the 70
———, ultimate vascularity of, remarks upon 93
Farre, Dr. case of cynanche laryngea 166
Fever, tropical, depletory method of cure, introduction of 76
———, brain, from intoxication, account of 77
———, remittent, of infants, symptoms of 238
———————, mode of curing 243
———, in the Mediterranean, account of 247
———, typhus, cured by antimony and calomel 322
Foulkes, Ann, particulars of her case 355, 482
Fourcroy, M. biographical account of 499
Galvanic battery, account of Mr. Children's 426
Gases, on the specific heat of 399
Gibbes, Dr. on the Melksham chalybeate 299, 383
Gibbon, Mr. on the case of Ann Foulkes 355
Gibney, Dr. on sea-bathing 224
Glasgow, graduates at the university of 347
Goderneau's powder, account of 222
Granger, Mr. explanation of his statement respecting Ann Moore 189
Hamilton, Mr. W. on mercury in obstinate vomiting 101
Hæmatirrhœa, fatal case of 339
Hæmatemesis, case of 513
Heart, diseases of 141, 157
Heat evolved during inflammation 323
———, intense, simple mode of obtaining 346
———, radiant, observations on 397
Hebb, Mr. C. H. translation of Corvisart's treatise on diseases of the heart 141
Hemingway, Mr. case of convulsions during labour 36
Henderson, Dr. address to the committee in Ann Moore's case 187
Hepatitis, case of 190
Herpetic affections cured by arsenic 23, 24, 26
Hernia cerebri, case of 74
Hydrophobia, utility of blood-letting in 19
Hydrosulphurets, observations on 256
Hyoscyamus, effects of, applied to the eye 70
Iron-moulds, method of taking out of cotton 84
Insanity, Mr. Lucett's mode of treating 125, 434
Institute, imperial, of France, proceedings of 251, 429, 517
INTELLIGENCE, medical and philosophical 79, 171, 248, 343, 424, 517
Intestines, schirrus of, from hairs in the canal 515
Jenner, Mr. description of his footstool (with a figure) 174
Johnson, Mr. on the influence of tropical climates 227
——————— on cold applications in ulcers 511
Jones, Mr. W. on puerperal convulsions 455
Juice, gastric, observations on 517
King, Mr. R. H. his case of small-pox after vaccination 33
Kinglake, Dr. on arsenic in herpetic affections 22, 29
——————— reply to Mr. Abel 96
Lamp for preventing explosions in coal-mines, account of 82
LECTURES, Dr. Squires', on midwifery, 174, 350. At Trinity College, Dublin, 261. Dr. Macartney's, on anatomy, ib. Dr. Merriman's, on midwifery, ib. 522. Dr. Clarke and Mr. Clarke's, ditto, ib. Mr. Taunton's, on anatomy, ib. Dr. Buxton's, on the practice of medicine, 262. Dr. Clough's, on midwifery, ib. Dr. Carpue's, on anatomy, ib. Mr. Brooke's, ditto, ib. Mr. Moor's, on diseases of the teeth, ib. Dr. Roget's, on the practice of physic, ib. Dr. Adams's, ditto, ib. Dr. Clutterbuck's, ditto, ib. Dr. Ramsbotham's, on midwifery, ib. 350. Dr. Dennison's, ditto, 347. Mr. Shute's, on anatomy, 348. Mr. Pettigrew's, ditto, ib.

LECTURES, at St. Thomas's and Guy's Hospitals, 347. By Mr. Cooper and Mr. Cline, Dr. Babington, Dr. Curry, Dr. Marcet, Dr. Cholmeley, Dr. Haighton, Mr. Allen, Mr. Fox.
———, at the London Hospital, ib. By Dr. Dennison. Mr. Brande, on chemistry, 348. Dr. George Pearson, on physic, &c. 350. Dr. Hooper and Dr. Ager, ditto, ib. Mr. Carlisle's, on surgery, ib. Mr. Brodie's, ditto, ib. Sir Everard Home's, ditto, ib. Mr. Stephenson's, on the eye and ear, 522.
Lepra, description of 418
———, vulgaris, cured by arsenic 267, 298
———, alphos, cured by arsenic 298
Lucett, Mr. account of his process of treating insanity 125, 434
Magendie, M. account of his memoir on vomiting 207
Magnesia, effects of, in preventing uric acid 324
Marcet, Dr. on chronic rheumatism 235
———, his mode of obtaining an intense heat 346
Marsh miasmata, effects of 15
Mayo, Mr. C. case of rupture of the uterus 444
Medical reform, observations on 2, 6, 177, 179, 265, 296, 385, 452
MEDICAL REPORT, half yearly 1
Medical topography of Nottinghamshire 99
Medical practitioners of Gloucestershire, resolutions of, in support of vaccination 257
——— of Britain, account of 266
——— practice in Scotland, state of 285
Melksham chalybeate and saline spa, account of 299, 383
Medicine, progress of 14
———, practice of, by the natives of India 220
Memorial addressed by Dr. Henderson to the committee on Ann Moore's case 187
Mercury, use of, in cases of vomiting 100
———, action of, in diseases 446
Merriman, Dr. S. on difficult parturition 163
METEOROLOGICAL TABLE, 87, 175, 263, 351, 428, 527
Mortification, occasioned by the application of a high temperature to parts torpid by cold 20
National vaccine establishment, report of 113
Natural history, importance of 13
Nausea and vomiting, distinction between 199
Nerves, chemical analysis of 67
New patents 85
Notices to correspondents 88, 176, 264, 352, 489, 528
Nottinghamshire, medical topography of 98
Ophthalmia, remarks on the cure of, by Mr. W. Adams 46
Opium, use of, in delirium tremens 17
———, in spasm of the uterus 29, 33
———, in labour pains 303
———, in constipation 442
Percival, Dr. E. on turpentine in epilepsy 509
Pericarditis, sub, case of 143
Pericardium, adhesion of, to the heart, case of 145
Peritoneal inflammation relieved by cold applications 19
Peritonitis, observations on 409
Picromel, a biliary substance, account of
Plants, sap of, described 251
Plates, Jenner's footstool 174
——— tumor on the neck 353
Playfair, Mr. G. cases of dysentery cured by ipecacuanha and laudanum 75
Portal, M. on the nature and treatment of apoplexy 193, 370
Pregnancy, abdominal, case of 318
Pring, Mr. D. on hernia cerebri 74
Prout, Dr. on the art of injecting blood-vessels 89
Psoriasis, diffusa, case of, cured by arsenic 297, 298
———, inveterata, ditto, ditto 297
———, scrotalis, ditto, ditto 299
Pulley, Mr. J. on the case of Ann Foulkes 481
Ramsbotham, Dr. J. on rupture of the uterus during labour 317
Rectum, contracted, case of 404
Report, medical, half yearly 1
——— of diseases 438, 527
——— of the committee for examining Messrs. Delahoyde and Lucett's process for curing insanity 433
——— of the national vaccine establishment 113
——— of the London committee of associated apothecaries 307
——— appendix to 387
——— botanical 524
Rheumatism, chronic, cured by warm clothing 235

Rheumatism, observations on the cure of 362
———, cases of 363, 367
Richerand, M. on gun-shot wounds 377, 458
Salivation, resuscitated, good effects of 37
Scot, Mr. W. on vaccination in India 506
Sea-bathing, observations on, by Dr. Gibney 224
Senter, Dr. case of urinous vomiting 493
Silt, description of 15
Small-pox, case of, after vaccination 33
Smith, Mr. T. on cold applications in inflammation 512
Society, royal, proceedings of 79, 248, 424
———, Linnæan, proceedings of 82, 250
———, medical, of Massachusetts, officers of 346
———, philosophical, of London 436
——— for the relief of widows and orphans of medical men 436, 521
Spark, Dr. on opium in spasm of the uterus 29-33
———, on spasm of the uterus 315
Splenitis, observations upon, by Dr. Bree 169
Stomach, cancerous, dissection of 337
———, affection of, from a reptile 514
Surrey institution 436
Sutliffe, Mr. on antimony and calomel in typhus 321
Sutton, Dr. on delirium tremens 17, 333
——— on the cure of rheumatism 362
——— on peritonitis 409
Table, meteorological 87, 175, 263, 351, 438, 527
——— of the annual mortality of the different counties of Great Britain for 1811 140
——— of drugs 437, 526
Tardy, Mr. on the treatment of insanity 125
Thenard, M. observations on hydrosulphurets 256
Thomson, Dr. T. on the heat evolved during inflammation 323
——— description of a resinous substance dug out of the earth at Highgate 343
——— biographical account of Fourcroy 499
Toulmin, Dr. on egg-like incrustations in urine 477
Turpentine, oil of, use of, in epilepsy 509
Vaccination, small-pox after 33
———, progress of 113
———, in India 216
Vaccination, in the isles of France and Bourbon 506
Vauquelin, M. analysis of the cerebral matter of man and other animals 55, 68
Vegetables, physiological observations on 252
Vomiting and nausea, distinction between 199
Vomiting, act of, explained physiologically 207
Ulcers, effects of cold applications in 511
University of Dublin, account of the 278
Universities of Scotland 287
Urine, egg-like appearances in 477
———, analysis of 234
Urinous vomiting, case of 465
———, remarks on 484
Uterus, rupture of, during labour 316, 444
Uwins, Dr. on constipation 442
Walcheren, island of, described 15
Walsh, Dr. E. case of hæmatirrhœa 339
Ward, Mr. James, account of Ann Moore and Mary Thomas 157
Wardrop, Mr. on albuminous concretions 73
Water, cold, effects of, in inflammation 512
Webster, Mr. reply to Mr. Granger 377
White, Mr. H. on arsenic in cutaneous complaints 296
——— on contracted intestinum rectum 403
Wishart, Mr. on congenital cataract 69
Wounds, gun-shot, on the nature and treatment of 377, 458
Wright, Mr. R. on medical reform 452
Yeats, Dr. on resuscitated salivation 37
——— on a case of ischuria 45
——— reply to Mr. Gibbon 197
——— explanation to ditto 488
Young, Dr. on the effects of climates 129
Zealand, noxious miasmata of 16

### PLATES.

Jenner's Footstool - - - - - 174
Tumor on the Neck - - - - - 353

*THE renewed intercourse with the Continent renders it proper to acquaint the friends of English Literature in those Countries to which the* MEDICAL AND PHYSICAL JOURNAL *is again accessible, that this work is regularly delivered by* THE POST-MASTERS, *in all parts of Europe, at* THREE GUINEAS *per annum, or* ONE GUINEA AND A HALF *for six months; and persons residing in England, desirous of having this Journal regularly delivered to any friend in any part of the world, may have the Numbers sent as published, on the same terms, by giving their orders, and making payment—*

*To* Mr. WILLIAM SERJEANT, *of the General Post Office, London, for the countries bordering on the Baltic and Mediterranean, and for Portugal and the Brazils.*

*To* Mr. COWIE, *G. P. O. for Hamburgh, Germany, and Holland.*

*To* Mr. THORNHILL, *G. P. O. for the West Indies, Bahama, Madeira, Bermuda, Canada, and Nova Scotia.*

*To* Mr. AUSTIN, *G. P. O. for Ireland.*

*And to* Mr. GUY, *of the India House, for the Cape, and all parts of India.*

*Nothing therefore is requisite but at the different places to give orders to the Post-masters, or in England to make payment to the persons above-named, or for them to any local Post-master, to secure the punctual and early delivery of this Journal in any part of the civilized world.*

